# Duodenal ulcer perforation presenting as acute appendicitis in a child: a case report and literature review

**DOI:** 10.3389/fsurg.2026.1764840

**Published:** 2026-06-02

**Authors:** Zhihui Jin, Xusheng Yang, Yuntian Liu, Bihui Yao, Xuewen Chen, Lu Liang

**Affiliations:** 1 Affiliated Baotou Clinical College of Inner Mongolia Medical University, Inner Mongolia, China; 2 Hepatobiliary Surgery Department, Baotou Central Hospital, Inner Mongolia, China; 3 National Key Clinical Specialty Development Project (General Surgery), Baotou Central Hospital, Baotou, China; 4 Second Affiliated Hospital of Baotou Medical College, Inner Mongolia, China

**Keywords:** acute appendicitis-like presentation, diagnostic laparoscopy, differential diagnosis, duodenal ulcer perforation, pediatric acute abdomen

## Abstract

**Introduction:**

Duodenal ulcer perforation (DUP) is exceedingly rare in the pediatric population. Its clinical presentation frequently mimics acute appendicitis (AA), leading to a high risk of preoperative misdiagnosis and posing substantial diagnostic and therapeutic challenges.

**Case presentation:**

We describe a 12-year-old boy who presented with signs and symptoms suggestive of acute appendicitis. Emergency diagnostic laparoscopy revealed a 0.6-cm perforation on the anterior wall of the duodenal bulb. The perforation was successfully repaired laparoscopically. The patient had an uneventful postoperative recovery, and follow-up endoscopy confirmed complete mucosal healing.

**Discussion:**

This case highlights that pneumoperitoneum in pediatric patients presenting with AA-like symptoms should prompt consideration of DUP in the differential diagnosis. Diagnostic laparoscopy is instrumental in establishing an accurate diagnosis and enabling timely, minimally invasive treatment.

**Conclusion:**

In children with atypical acute abdominal pain, heightened clinical suspicion for DUP, particularly when imaging reveals pneumoperitoneum, combined with proactive use of laparoscopy, can reduce misdiagnosis and improve patient outcomes.

## Introduction

Peptic ulcer disease (PUD) is uncommon in children, with reported prevalence rates of approximately 8.1% in Europe and an annual incidence of 1.55 cases per year in India. In pediatric patients with duodenal ulcers, perforation rates range from 0% to 9%, markedly lower than those observed in adults (1, 2). Moreover, the etiology of pediatric PUD differs from that in adults, demonstrating a weaker association with *Helicobacter pylori* infection and non-steroidal anti-inflammatory drug (NSAID) exposure (3). When DUP occurs, leakage of gastric or duodenal contents into the peritoneal cavity may result in diffuse peritonitis, a serious condition if not promptly recognized and treated. In children, the clinical manifestations of DUP are often nonspecific and may mimic other acute abdominal conditions. Migration of leaked gastrointestinal fluids along the right paracolic gutter to the right lower quadrant can produce pain and localized tenderness similar to acute appendicitis (AA), contributing to preoperative misdiagnosis (4). Imaging plays a pivotal role in evaluation, with free subdiaphragmatic air on abdominal computed tomography (CT) serving as a key indicator of gastrointestinal perforation. When pneumoperitoneum (The presence of free air in the peritoneal cavity, a key radiological sign of gastrointestinal perforation) is detected preoperatively, careful interpretation of radiologic findings is essential to avoid diagnostic error. Misdiagnosis may lead to inappropriate operative approaches and delayed definitive management, thereby increasing the risk of postoperative complications. This case report details the diagnostic and therapeutic course of a 12-year-old boy initially diagnosed with AA who was ultimately found to have DUP during laparoscopy. We examine the factors contributing to misdiagnosis, highlight the diagnostic value of radiological clues, and discuss the essential role of diagnostic laparoscopy in managing such complex presentations. Laparoscopy offers a comprehensive field of view for detecting subtle perforations and allows definitive repair during the same procedure, thereby reducing surgical trauma and expediting recovery (5).

This report was prepared in accordance with the SCARE 2023 guidelines (6).

## Case presentation

**Figure 1:**
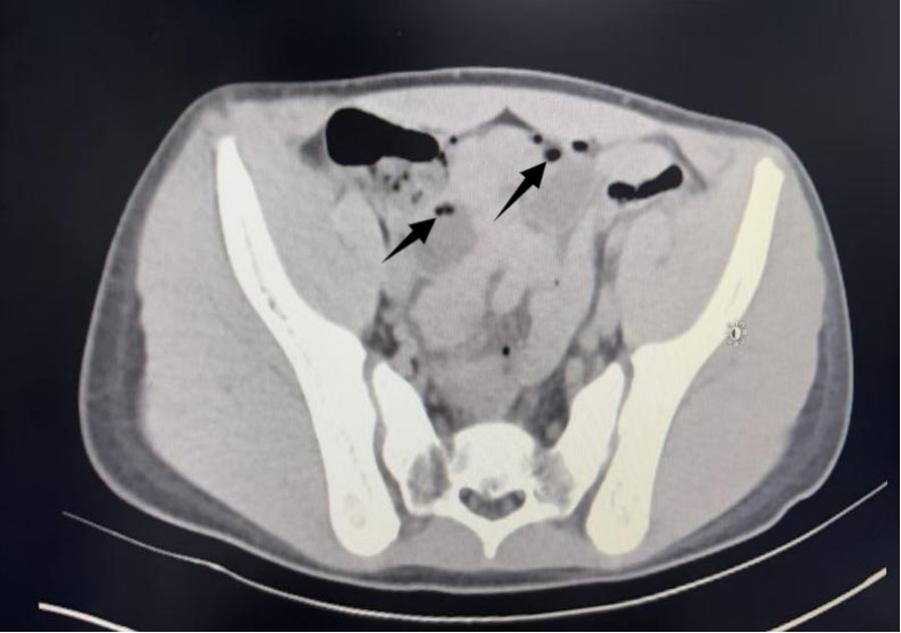
Preoperative pelvic CT showing free intraperitoneal air.

**Figure 2:**
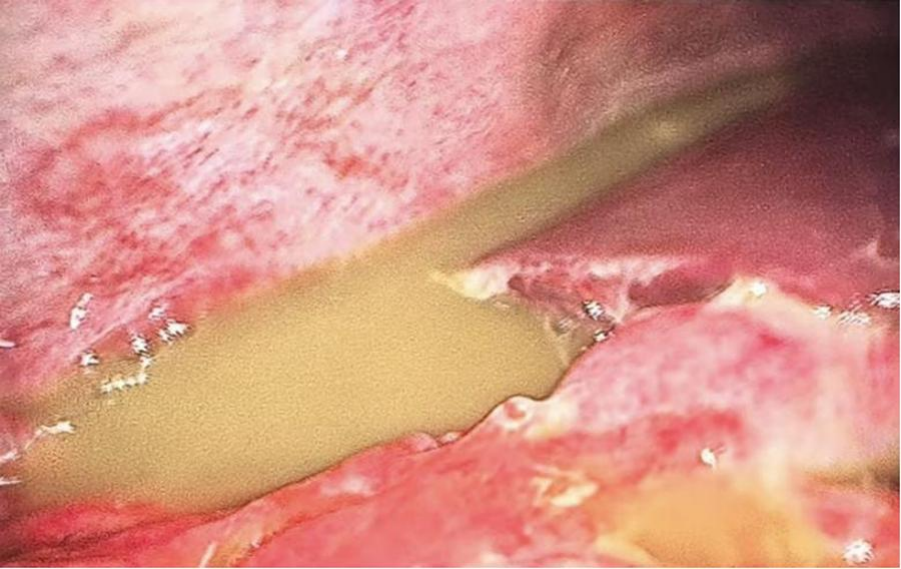
Intraoperative findings demonstrating a large volume of yellow-brown purulent fluid within the peritoneal cavity.

**Figure 3:**
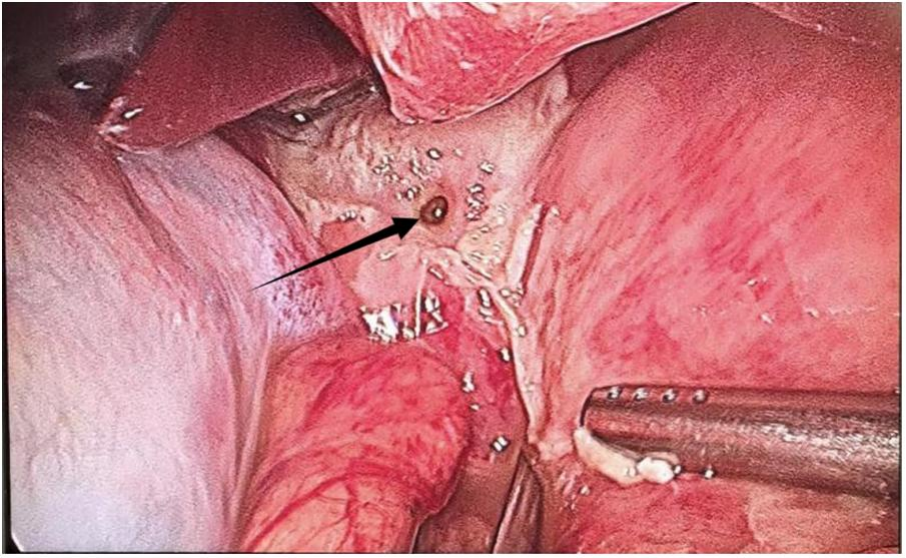
Intraoperative visualization of a 0.6-cm perforation on the anterior wall of the duodenal bulb.

A 12-year-old boy presented with a sudden onset of epigastric pain one day before admission, without any identifiable precipitating factors. The pain later migrated and localized to the right lower quadrant and progressively intensified. He reported nausea and a single episode of non-bilious, gastric-content vomiting. There was no history of fever, chills, or diarrhea. According to the guardian, the patient had no prior abdominal pain or regular medication use, the patient was hemodynamically stable with normal vital signs, despite signs of generalized peritonitis. On physical examination, he was alert and fully oriented. Abdominal assessment revealed board-like rigidity and diffuse tenderness, most pronounced in the right lower quadrant along with marked rebound tenderness at McBurney's point. He had no prior episodes of similar symptoms and no relevant family history. Laboratory evaluation showed a white blood cell count of 11.08 × 10^9^/L, with 93.8% neutrophils (absolute neutrophil count = 10.04 × 10^9^/L). Abdominal ultrasonography demonstrated an enlarged appendix (outer diameter = 0.73 cm) and free intraperitoneal fluid. Contrast-enhanced CT of the lower abdomen and pelvis revealed pneumoperitoneum and pelvic effusion, raising suspicion for a hollow viscus perforation, with appendiceal perforation not excluded but failing to visualize the upper abdominal duodenum due to restricted scanning range (Figure 1). For pediatric patients with classic clinical features suggestive of acute appendicitis, limited CT imaging of the symptomatic right lower quadrant is the first-line imaging modality, chosen to minimize unnecessary radiation exposure to the chest and upper abdominal regions. A provisional diagnosis of perforated AA with diffuse peritonitis was made, and emergency laparoscopic exploration was undertaken. Intraoperatively, approximately 100 mL of yellow-brown purulent fluid was identified within the peritoneal cavity (Figure 2). Systematic exploration of the appendix, ileocecal region, and small intestine revealed no significant abnormalities. Further evaluation of the upper abdomen showed inflammatory adhesions between the duodenal bulb and the inferior surface of the liver, with fibrinous exudate coating the surrounding tissues. Following careful adhesiolysis, a 0.6-cm perforation was visualized on the anterior wall of the duodenal bulb, with active leakage of gastrointestinal contents (Figure 3).

**Figure 4:**
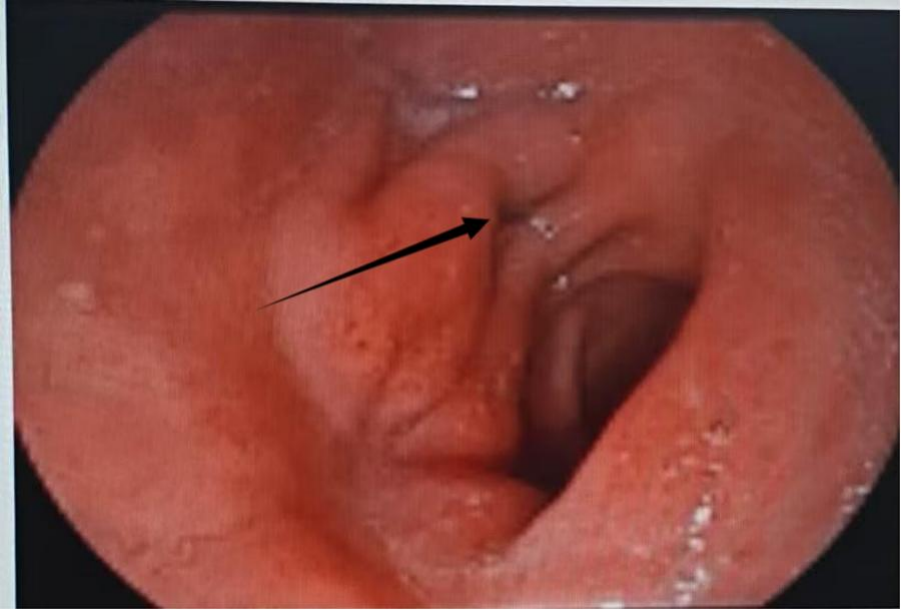
Postoperative follow-up gastroscopy showing satisfactory healing of the duodenal bulb ulcer scar.

Based on these findings, a definitive diagnosis of DUP was established. A modified laparoscopic Graham Patch repair was performed. An abdominal drainage tube was inserted through the foramen of Winslow, externalized via a stab incision on the right abdominal wall, and securely fixed in position. All incisions were closed in layers. The postoperative course was uneventful. The patient passed flatus on postoperative day (POD) 3, and the intra-abdominal drainage catheter was removed on POD 5. He was discharged on POD 7 with instructions to continue oral proton pump inhibitor therapy, gastric mucosal protective agents, and empirical *H. pylori* eradication therapy for 14 days. The guardian expressed satisfaction with minimally invasive laparoscopic treatment and confirmed the patient's complete return to normal diet, school, and daily activities 1 month after discharge. Follow-up gastroscopy at one month revealed deformity of the duodenal bulb with a well-healed round scar at the prior perforation site. The surrounding mucosa was congested and edematous, while the descending duodenum was unremarkable, consistent with satisfactory ulcer healing (Figure 4) and the ¹³C-labeled urea breath test was negative. At the 3- and 6-month follow-ups, the patient had no recurrence of abdominal pain, nausea, vomiting or other digestive symptoms. No postoperative complications such as intestinal adhesion, ulcer recurrence or abdominal infection were noted.

## Discussion

DUP is exceedingly rare among pediatric patients presenting with acute abdominal emergencies, accounting for well under 1% of cases (7). The classic clinical presentation consists of sudden onset of severe epigastric pain, signs of peritoneal irritation, and radiographic evidence of subdiaphragmatic free air on abdominal x-ray or CT. However, because children often have difficulty describing their symptoms accurately and clinicians encounter this condition infrequently, DUP is seldom considered during the initial diagnostic evaluation. As a result, it is frequently misdiagnosed as more common causes of acute abdomen, particularly AA, leading to delayed or incorrect diagnosis. This clinical presentation is known as Valentino's Syndrome, a rare condition in which duodenal perforation manifests with right lower quadrant pain mimicking acute appendicitis. This diagnostic challenge was clearly demonstrated in the present case. The patient's preoperative symptoms, laboratory findings, and even ultrasonographic features strongly supported a diagnosis of perforated acute appendicitis, illustrating a classic diagnostic trap in pediatric emergency care.

The differential diagnosis also includes gastrointestinal perforation secondary to foreign body ingestion. Foreign body perforation requires a clear history of ingestion and corresponding radiologic signs, which were absent in this patient. Ovarian torsion was excluded because the patient was male; mesenteric lymphadenitis was also ruled out, as the patient had no fever and CT imaging showed no significant lymphadenopathy. Perforation related to congenital anomalies such as intestinal atresia or Hirschsprung disease typically presents in the neonatal period or early infancy. CT imaging usually demonstrates dilated bowel loops or features of obstruction indicative of underlying developmental abnormalities. Such conditions are highly improbable in an previously healthy 12-year-old child, and no radiologic evidence supported these diagnoses (10).

Intraoperative exploration revealed no abnormalities of the appendix or ileocecal region; instead, a perforation was identified on the anterior wall of the duodenal bulb, confirming the diagnosis of DUP. This finding highlights the subtle clinical presentation and substantial diagnostic challenges associated with pediatric DUP (3). Pediatric PUD can be classified as primary, most commonly related to *Helicobacter pylori* infection, or secondary, typically arising from physiological stress, systemic illness, or the use of NSAIDs (1). In a retrospective study by Yeh et al. (8), approximately half of pediatric patients with duodenal ulcers were negative for *H. pylori* and had no history of NSAID exposure; however, evidence on idiopathic duodenal ulcers in children remains limited. The patient in our case followed this pattern, with neither preceding NSAID use nor a history of *H. pylori* infection.

The misdiagnosis of DUP as AA in this case was primarily attributable to the substantial overlap in clinical manifestations. The underlying pathophysiology involves leakage of gastric and duodenal contents, which track along the right paracolic gutter into the right lower quadrant and pelvis. This chemical irritation preferentially stimulates the parietal peritoneum in the right lower abdomen, altering pain localization and producing classic McBurney's point tenderness. Inflammatory mesenteric edema may further mimic appendiceal pathology on imaging, leading to mild appendiceal enlargement and generating a diagnostically misleading “false positive”. In addition, the imaging examinations had the following limitations: The limited lower abdominal CT detected pneumoperitoneum and pelvic effusion but failed to visualize the upper abdomen. restricted imaging field-combined with pediatric factors such as small body habitus, excessive bowel gas, and reduced spatial resolution, limited the ability to accurately identify the perforation site. Third, duodenal ulcer perforation (DUP) is exceedingly rare in children, and was therefore not included in the initial differential diagnosis in the differential diagnosis for AA-like symptoms. This case underscores a crucial diagnostic principle. Notably, when AA is suspected clinically, but imaging shows pneumoperitoneum, an atypical sign for appendicitis, upper gastrointestinal perforation should be strongly considered (10). When available, a whole-abdomen CT scan can increase diagnostic accuracy and help locate the perforation site. In pediatric patients presenting with classic clinical features of acute appendicitis and no atypical radiological signs, limited CT imaging of the symptomatic region remains the preferred modality, as it effectively minimizes unnecessary radiation exposure. In such complex diagnostic scenarios, laparoscopic exploration is particularly valuable. It provides a comprehensive, magnified view of the entire abdominal cavity, enabling direct identification of the underlying pathology and allowing definitive minimally invasive repair within the same procedure. This combined diagnostic and therapeutic capability avoids unnecessary laparotomy, reduces delays associated with misdiagnosis, and has established laparoscopy as an essential modality for managing complicated acute abdominal conditions by minimizing surgical trauma and expediting recovery (9).

The therapeutic strategy in this case combined precise minimally invasive surgery with comprehensive perioperative management. Surgically, for DUPs <1 cm with viable tissue margins, the modified laparoscopic Graham patch is widely accepted as a safe and effective standard surgical technique for this patient group (9, 11). This modified approach provides superior mechanical reinforcement and biological sealing compared with the classic Graham patch, an advantage that is especially relevant in pediatric patients given their smaller abdominal cavities and more delicate gastrointestinal tissue (11, 12). Laparoscopic surgery offers minimal trauma, a broad surgical view, and rapid recovery while achieving reliable tissue reinforcement and sealing to promote ulcer healing. Open surgery was not considered because it involves larger incisions, longer recovery, and a higher risk of intestinal adhesion in children. Perioperative management is equally critical. Sustained acid suppression with proton pump inhibitors (PPIs), appropriate antimicrobial therapy, and early enteral nutrition constitute key components that support postoperative recovery and optimize mucosal healing (13). Idiopathic duodenal ulcer is the most common form in children. However, Helicobacter pylori infection also warrants clinical attention, as it represents an important etiological factor for DU in the pediatric population (8). Given the emergent presentation of this case, preoperative H. pylori testing was not feasible; thus, empirical anti-H. pylori therapy was initiated in accordance with the 2023 ESPGHAN/NASPGHAN clinical guidelines for the management of H. pylori infection in children and adolescents (14), which recommend empirical eradication therapy for pediatric duodenal ulcer perforation when preoperative diagnostic testing cannot be performed, to reduce the risk of ulcer recurrence. Moreover, noninvasive *H. pylori* testing (¹³C-urea breath test or stool antigen assay) should be performed 4–8 weeks after treatment completion to confirm eradication and to avoid unnecessary antibiotic exposure.

Comparison with previously reported pediatric DUP cases reveals several shared characteristics and important distinctions (Table 1). Historically, most pediatric DUP cases were diagnosed only after the onset of severe peritonitis and often required open exploratory laparotomy for confirmation (16). In contrast, our case is one of the few diagnosed and treated via laparoscopic exploration, similar to the report by Morrison et al. (15), illustrates the dual diagnostic and therapeutic advantages of laparoscopy in evaluating complex acute abdominal presentations. Laparoscopy played a pivotal role, enabling prompt diagnosis and repair despite the initial misdiagnosis, and was associated with shorter hospital stay and better pain control compared to laparotomy. Similar misdiagnoses have been reported, and pneumoperitoneum on imaging served as a key clue to distinguish perforated ulcer from appendicitis (15). This case is unique in its typical Valentino's Syndrome presentation and complete long-term follow-up evidence chain, which provides more reliable clinical evidence for avoiding misdiagnosis and minimally invasive management. In summary, this rare case not only enriches the current literature on pediatric DUP with atypical acute appendicitis-like symptoms, but also reinforces the clinical value of laparoscopic exploration in enhancing diagnostic accuracy and optimizing minimally invasive treatment for such challenging pediatric acute abdomen.

**Table 1:** Comparison of clinical characteristics in reported pediatric cases of DUP.

Author (year/country)	Age	Sex	Diagnosis	Diagnostic method	Perforation site	Perforation size (cm)	Surgical procedure	Outcome	Postoperative Follow-up Findings
Hua et al. (3) (2007/US)	14	M	DUP	Diagnostic laparoscopy	Duodenal bulb	0.4 × 0.4	Modified laparoscopic Graham Patch	Discharged on postoperative day 7 with uneventful recovery	No additional follow-up data available
Hua et al. (3) (2007/US)	13	F	DUP	Diagnostic laparoscopy	Duodenal bulb	2 × 1	Gastroduodenostomy with paraduodenal drainage placement	Discharged on POD 40 with a smooth postoperative course	Recovered well without any complications at follow-up
Shrestha and Shrestha (4) (2021/Nepal)	12	F	Peritonitis	Exploratory laparotomy	Duodenal bulb	0.2 × 0.3	Graham omental patch repair+FJ	Discharged on postoperative day 11 with uneventful recovery	Helicobacter pylori negative at 1-week follow-up
Suraj et al. (10) (2025/Nepal)	9	M	DUP	Exploratory laparotomy	Duodenal bulb	0.5 × 0.5	Graham omental patch repair	Discharged on postoperative day 6 with uneventful recovery	No complications at 2-week review; regular diet resumed at 1 month
Morrison et al. (15) (2013/US)	9	F	DUP	Diagnostic laparoscopy	Duodenal bulb	0.3 × 0.3	Modified laparoscopic Graham Patch	Discharged on postoperative day 7 with uneventful recovery	Gastroscopy at 6 weeks confirmed H. pylori eradication; negative mucosal biopsy verified infection clearance
Getu et al. (16) (2025/Ethiopia)	4	M	Malaria, DUP	Exploratory laparotomy	Duodenal bulb	2 × 2	Graham omental patch repair	Discharged on postoperative day 7 with uneventful recovery	No complications and negative H. pylori at 1-week follow-up

F, female; M, male; DUP, duodenal ulcer perforation; FJ, feeding jejunostomy.

## Conclusion

In summary, DUP in children is exceedingly rare. This report describes a particularly deceptive preoperative presentation and an intraoperatively confirmed DUP, supported by a complete diagnostic and follow-up evidence chain, including laboratory testing, ultrasonography, CT imaging, intraoperative laparoscopic findings, and postoperative endoscopic evaluation. These data provide a valuable clinical reference for the management of uncommon pediatric abdominal emergencies. This case highlights the importance of a broad differential diagnosis for children with acute abdominal pain. Clinicians must remain vigilant to the possibility of rare but serious conditions that may masquerade as more common pathologies, and should carefully scrutinize all radiological findings, particularly when they appear discordant with the clinical picture. When discrepancies exist between clinical manifestations and auxiliary examinations, diagnostic laparoscopy is strongly recommended as a first-line approach. By integrating accurate diagnosis with timely treatment, laparoscopy helps reduce misdiagnosis, facilitate minimally invasive intervention, and optimize clinical outcomes in pediatric patients.

## Data Availability

The raw data supporting the conclusions of this article will be made available by the authors, without undue reservation.
